# Preventive effects of inotodiol on polyinosinic–polycytidylic acid-induced inflammation in human dermal fibroblasts

**DOI:** 10.1016/j.heliyon.2023.e20556

**Published:** 2023-10-10

**Authors:** Gun-Woo Won, Seung Hoon Lee, Mahesh Prakash Bhatta, Seung-Hyeon Choi, Cheong-Hae Oh, Jong-Tae Park, Jong-Il Park

**Affiliations:** a Department of Biochemistry, College of Medicine, Chungnam National University, Daejeon, 35015, Republic of Korea; b Translational Immunology Institute, Chungnam National University College of Medicine, Daejeon, Republic of Korea; c BK 21 FOUR, Chungnam National University Department of Medical Science, Daejeon, Republic of Korea; d Department of Food Science and Technology, Chungnam National University, Daejeon, 34134, Republic of Korea; e CARBOEXPERT Inc., Daejeon, 34134, Republic of Korea

**Keywords:** Inotodiol, Poly(I:C), HDFs, Anti-inflammation, Toll-like receptor 3 signaling pathway

## Abstract

Double-strand RNA(dsRNA), which can induce inflammation, can be generated by necrotic keratinocytes in the skin environment. As an analog of dsRNA, polyinosinic–polycytidylic acid (poly(I:C)) is used to induce inflammation via the Toll-like Receptor 3 (TLR3) signaling pathway. Inotodiol, isolated from Inonotus obliquus, known as Chaga mushroom, is a natural lanostane-type triterpenoid with significant pharmacological activity and notable anti-inflammatory effects. However, the functions of inotodiol on dsRNA-induced inflammation in human dermal fibroblast (HDFs) remains unclear. In this study, we evaluated the anti-inflammatory effects of inotodiol inflammation induced on by poly(I:C) in HDFs. After pre-treatment with inotodiol, poly (I:C) was used to induce inflammation. Subsequently, mRNA expression and protein secretion of inflammatory cytokines, as well as TLR3 signaling protein levels were assessed. Inflammatory cytokines IL-1β, IL-6, and TNF-α′s increased mRNA expression by poly(I:C) in HDFs was significantly suppressed in the inotodiol pre-treatment group in a dose-dependent manner. A similar pattern was evaluated in the protein levels of these three cytokines. The inflammatory signals of TLR3 via *p*-IKK, p-p38, and NF-κB was reduced by inotodiol pre-treatment. Taken together, inotodiol possesses strong anti-inflammatory activity against poly(I:C)-induced inflammation in HDFs. Therefore, our findings support potential application of inotodiol as an effective anti-inflammatory agent in cosmetics.

## 1 Introduction

The skin is a large organ that acts as an effective barrier over the body surface and accounts for 16 % of the body mass [1]. It is composed of an outer epidermis layer and an inner dermal layer [2]. The skin protects the soft internal tissues from physical damage, bacterial infection, and dehydration [1]. The dermal layer plays a role in supporting the epidermis layer, and it is known that skin elasticity decreases because of aging, inflammation, and dryness [2,3]. The dermal layer consists of connective tissue and is rich in fibroblasts [4]. These fibroblasts secrete collagen, an extracellular matrix component, which induces inflammation by secreting inflammatory cytokines when exposed to bacteria and viruses [5]. The dermal layer can induce inflammation in damaged cells, causing exposure of the epidermal layer to biotic and antibiotic factors [[6], [7], [8]].

In addition, dsRNA is generated by infected cells during the replication of dsRNA viruses, which binds with TLR3 leading to a strong anti-viral response in host cells [9]. Cellular dsRNA can be produced by uninfected cells, such as apoptotic, necrotic, or otherwise stressed cells [10]. Moreover, previous studies demonstrated that dsRNA is caused and produced by noncoding RNA from UV-irradiation damages in keratinocytes, triggering skin inflammatory reactions, which in turn induces the expression of inflammatory cytokines, via TLR3 signal pathways, such as IL-6 and TNF-α in non-irradiated cells [8].

The Pathogen-Associated Molecular Pattern (PAMP), is related to inflammation caused by skin damage and invasion by microorganisms [11]. Among these, TLR3 was originally found to recognize polylysine-polycytidylic acid (poly(I:C)), a synthetic analog of double-stranded RNA, and it is crucial for the immune system's defense against viral infection [12]. Poly(I:C) treatment mimics the viral infection and induces the secretion of type 1 interferons (IFNs) and inflammatory cytokines as an anti-viral immune response [13,14]. This immune response is mediated by the TLR3 pathway, and when activated, the signal is transduced to NF-κB and the expression of IFNs induces cell regeneration [15,16].

**Fig. 1:**
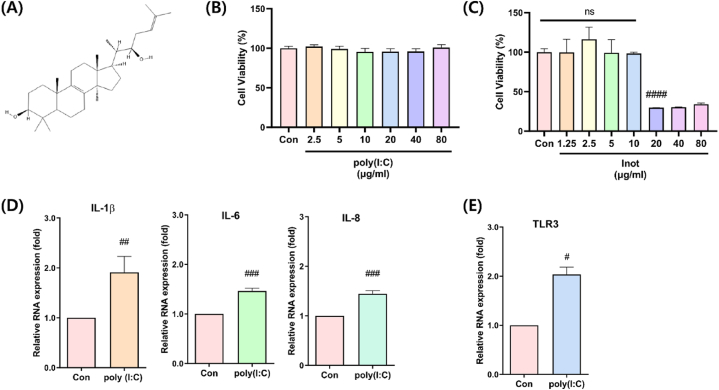
Measurement of inotodiol cytotoxicity and establishment of polyIC-induced inflammation model. (A) Chemical structure schematic diagram of inotodiol. (B) Poly(I:C) was treated at each concentration of 2.5–80 μg/ml for 24 h and cytotoxicity was measured, (C) In HDF cells, inotodiol was treated with culture media for 24 h, 1.25–80 μg/ml concentration and cytotoxicity was measured using WST-8 assay. (D) Poly(I:C) was treated for 24 h, and RNA levels of IL-1beta, IL-6, IL-8, and (E) TLR3 were analyzed by qRT-PCR after RNA isolation. Data are presented in mean values ± SD. *p < 0.05; ****p < 0.0001 vs. the control group.

Chaga mushroom (Inonotus obliquus) extracts are used in traditional medicine as they are considered effective in treating inflammation and atopy and reducing cholesterol and possess antioxidant properties [[17], [18], [19], [20], [21]]. Inotodiol, a lanostane triterpenoid and a component of Chaga mushroom extracts (Fig. 1A), has been reported to have an anti-allergic effect that selectively inhibits inflammation in mast cells and induces maturation of dendritic cells [[22], [23], [24]]. In addition, inotodiol inhibits cell proliferation and migration in melanocytes, and it has been reported that it inhibits cell migration and invasion and induces apoptosis in HeLa cell lines [[25], [26], [27], [28]].

In this study, inflammation was induced using poly(I:C) in human dermal fibroblast primary cells after pre-treatment with inotodiol to determine its anti-inflammatory effect and prevent functional damage caused by inflammation. Furthermore, we attempted to determine the differences in signal transduction pathways for the anti-inflammatory effect of inotodiol. The results suggest that inotodiol is effective in preventing inflammation in human dermal fibroblasts (HDFs).

## 2 Materials and methods

### 2.1 Material and reagents

The inotodiol powder was supplied from CARBOEXPERT Inc.,(Daejeon, Republic of Korea) and dissolved in 100 % pure ethanol, and then store at 4 °C before use. Chaga mushroom powder was purchased from Jungwoodang Co., Ltd. (Seoul, Republic of Korea) for Inotodiol extraction and purification, and quality analysis was performed by CarboExpert Inc., according to a previously reported protocol [29].

### 2.2 Cell culture and poly(I:C) stimulation

The Chungnam National University Hospital provided the human dermal fibroblast cells, which were obtained from plastic surgery patients. HDF cells were cultured in Dubelcco's modified Eagle's medium(DMEM; Welgene, Gyeongsan, Korea), supplemented with 10 % fetal bovine serum and 1 % antibiotic solution (PSG; GibcoBRL, Grand Island, NY, USA). A humidified atmosphere with 5 % CO2 (v/v) was used to culture the cells in a 100 mm culture dish at 37 °C. The medium was changed Every 3 days. After being detached with trypsin/EDTA, HDF cells were sub-cultured before being seeded in 96-well and 6-well plates to perform tests. The culture medium was changed to serum-free medium and maintained for 24–48 h after the end of the experiment in order to induce poly(I:C) stimulation. The ethics statement received approval from the Institutional Review Board (IRB) of Chungnam National University Hospital (IRB No. 2020-11-001), and the research was performed in accordance with the given guidelines.

### 2.3 Cell viability

Cell Counting Kit-8 (CCK-8) test (CCK-8, Biomax, Seoul, Korea), which detects dehydrogenase activity in the presence of an electron mediator, was used to measure the vitality of the cells. The HDF cells were treated with various amounts of poly(I:C) and inotodiol after being cultured in 96-well plates at a density of 2000 cells per well. The plates were incubated for another 24 h in the incubator at 37 °C and 5 % CO2. Next, 10 L of the CCK-8 solution was added to each 96-well plate well and incubated for one to 4 h. A microplate reader (PerkinElmer, Massachusetts, USA) was used to measure absorbance at 450 nm.

### 2.4 RNA isolation and quantitative RT-PCR

Total RNA was isolated from the HDF cell using the TRIzol reagent according to the manufacturer's instructions (Thermo Fisher Scientific, Massachusetts, USA). Complementary DNA (cDNA) synthesis was performed using the AccuPower RT PreMix according to the manufacturer's guidelines (Bioneer, Daejeon, Korea). The cDNA was amplified using specified primers (Table 1). A T100 Thermal Cycler (Bio-Rad, California, USA) was used for reverse transcriptional Polymerase chain reaction (PCR) cDNA synthesis. A Bio-Rad CFX Connect Real-Time PCR Detection System with SYBR Green Master Mix (Applied Biosystems, Massachusetts, USA) was used to measure mRNA expression. All PCR assays were performed in triplicates. The data on relative gene expression was examined using the 1−ΔΔCt method.

**Table 1:** PCR primer List of pro-inflammatory genes.

Primer name		Primer sequence (5′-3′)	Product length
h IL-1β	Forward primer	AAACAGATGAAGTGCTCCTTCCAGG	391 bp
	Reverse primer	TGGAGAACACCACTTGTTGCTCCA	
h IL-6	Forward primer	ATGAACTCCTTCTCCACAAGC	264 bp
	Reverse primer	GTTTTCTGCCAGTGCCTCTTTG	
h IL-8	Forward primer	ATGACTTCCAAGCTGGCCGTGGCT	293 bp
	Reverse primer	TCTCAGCCCTCTTCAAAAACTTCTC	
h TNF-α	Forward primer	GAGCTGAGAGATAACCAGCTGGTG	237 bp
	Reverse primer	CAGATAGATGGGCTCATACCAGGG	
h Actin	Forward primer	GGCGGACTATGACTTAGTTG	236 bp
	Reverse primer	AACAACAATGTGCAATCAA	

### 2.5 Western blotting

The HDF cells were lysed in Pro-Prep (Intron, Daejeon, Korea) lysis buffer before being centrifuged at 600×*g*, for 30 min with a cell lysate containing equivalent quantities of protein. Total protein was determined using a Quick Start Bradford 1× dye reagent (Bio-Rad, California, USA). Protein samples were loaded onto SDS-polyacrylamide gels and then transferred to PVDF membranes. Membranes were blocked for 1 h at room temperature in 3 % skim milk and 5%BSA (0.1 % Tween-20 TBS buffer). The membranes were treated overnight at 4 °C with primary antibodies. The antibodies used were as follows: anti-actin antibody was purchased from Santa Cruz Biotechnology, while antibodies against *p*-IKK, NF-κB, p–NF–κB, *p*-IκB, and p-p38 were purchased from Cell Signaling Technology. Secondary antibodies of Goat anti-rabbit and goat anti-mouse were obtained from Santa Cruz Biotechnology. Following a 1 h TBS-T wash, membranes were incubated for 1 h at room temperature with a secondary antibody (anti-mouse or anti-rabbit IgG-HRP, peroxidase conjugate, 1:2000 dilution in TBS-T) and cleaned with TBS-T for 1 h. Using ECL® reagents, bound peroxidase was detected (Millipore, Billerica, MA, USA).

### 2.6 ELISA

HDF cells were treated with poly(I:C) at a concentration of 10 μg/ml, in 96-well plates. After 24 h, the supernatants were collected, and an ELISA (Thermo Fischer scientific ELISA system (R&D Systems)) was used to detect the levels of cytokines, including IL-1, IL-6, and TNF- α.

### 2.7 Statistical analysis

Data are presented as mean ± standard error (SEM). A one-way analysis of variance (ANOVA). GraphPad Prism v.8.0.1 software was used to analyze all the data (GraphPad Software Inc., San Diego, CA, USA). One-way ANOVA with the Tukey multiple comparison post-test was used to compare statistics among various treatments. A significance level of p < 0.05 was considered statistically significant.

## 3 Results

### 3.1 Effects of inotodiol and poly(I:C) on the viability of HDF cells

We first performed cytotoxicity measurement assays to determine the appropriate concentrations of inotodiol and poly(I:C) in HDFs. Treatment with poly (I:C) at concentrations of 0–80 μg/mL did not induce cytotoxicity (Fig. 1, B). In addition, to determine the cytotoxicity of inotodiol, HDF cells were treated for 24 h with 0–80 μg/mL. The cytotoxicity of inotodiol was approximately 70 % at a dosage of 20 μg/mL or higher (Fig. 1, C). To further investigate the conditions of inflammation induction using poly(I:C), qRT-PCR analysis was performed after poly(I:C) treatment for 24 h to determine the RNA expression level of the inflammatory markers IL-1β, IL-6, and IL-8. We found that the expression levels of IL-1β, IL-6, and IL-8 were in the poly(I:C) treatment group were considerably higher than that in control group (Fig. 1, D). Furthermore, we evaluated TLR3 expression in the poly(I:C) treatment group (Fig. 1, E). We next induced inflammation in HDFs under these conditions.

### 3.2 Inotodiol reduced poly(I:C)-induced inflammation in HDF cells

**Fig. 2:**
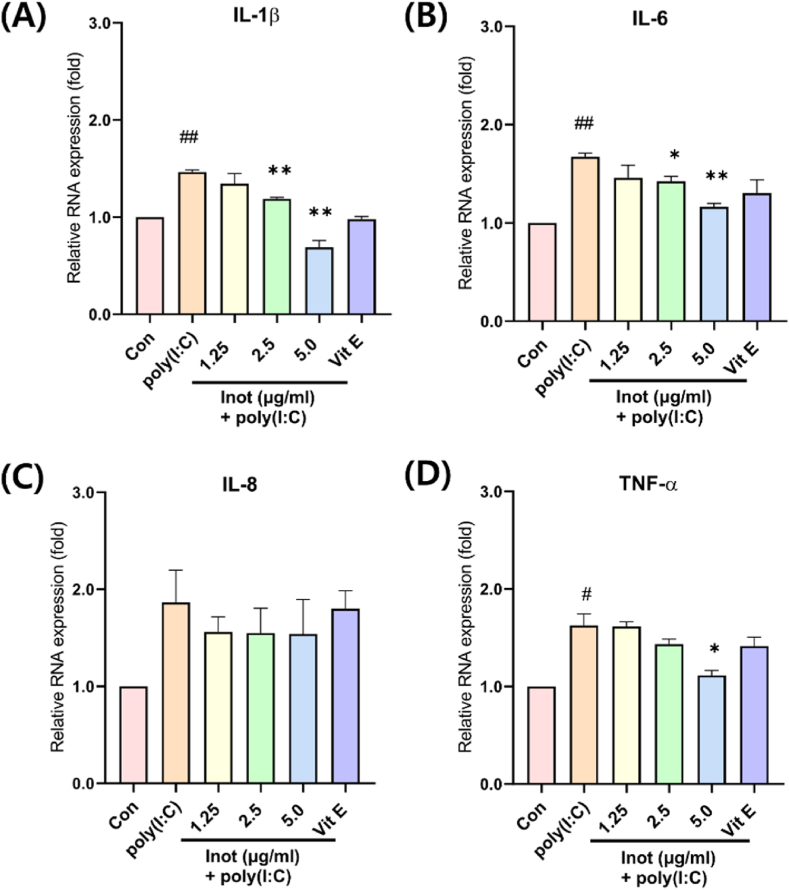
Poly(I:C)-induced inflammation was reduced dose-dependently by pretreatment with inotodiol in HDF cells. HDF cells were pre-treated with notodiol dose-dependently with serum-free media 5hr and incubated poly(I:C) 10 μg/ml for 24 h and qRT-PCR analysis. (A) shown RNA Level of IL-1β, (B) IL-6, (C) IL-8, (D) TNF-α. In addition, pretreated with Vitamin E (50μM), as A positive control of the anti-inflammation effect. Data are presented in mean values ± SD. #p < 0.05; ##p < 0.005 vs. the control group. *p < 0.05; **p < 0.005 vs. the poly(I:C) group.

To investigate the potential preventive effect of inotodiol on inflammation, we examined the RNA levels of cytokines. In addition, as a positive control for the anti-inflammatory effect, we used Vitamin E at a concentration of 50 μM. The mRNA levels of IL-1β, IL-6, and TNF-α (except for IL-8) were decreased in the inotodiol pre-treated cell group (Fig. 2). The results suggest that inotodiol inhibits RNA expression of IL-1β, IL-6, and TNF-α genes. In addition, poly (I:C)-induced inflammation decreased in an inotodiol dose-dependent manner. These results suggest that the inotodiol anti-inflammatory effects directly affected the inflammatory pathway.

**Fig. 3:**
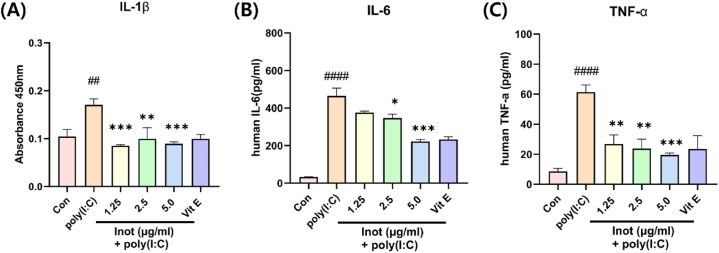
Inflammation-induced by poly (I:C), inotodiol pre-treatment reduced the secreted inflammatory marker proteins. HDF cell was pre-treated with Inotodiol dose-dependently with serum-free media 5 h and incubated poly(I:C) 10 μg/ml for 24 h and ELISA analysis. (A) shown protein Levels of IL-1β containing media (B) indicated IL-6 levels also (C) indicated TNF-α. Data are presented in mean values ± SD. ##p < 0.005; ####p < 0.0001 vs. the control group. *p < 0.05; **p < 0.005; ***p < 0.0005 vs. the poly(I:C) group.

*3.3 Inotodiol decreased secretion of inflammatory cytokines induced by poly(I:C) in HDF cells* In previous investigations, we demonstrated that pre-treatment with inotodiol in poly(I:C)-induced inflammation decreased the mRNA expression of inflammatory genes in a dose-dependent manner. To further examine whether the expression of the inflammatory protein secreted from HDF was also reduced by inotodiol, we performed an ELISA assay to measure the amount of IL-1β, IL-6, and TNF-α secreted in the media. As expected, the inflammatory cytokine secretion was considerably reduced in a dose-dependent manner (Fig. 3); this decrease indicates an anti-inflammatory effect, reinforcing previous results. These findings indicate that inotodiol prevented poly(I:C)-induced inflammation by inhibiting protein secretion and thereby reduced gene expression.

### 3.3 Inotodiol reduced poly(I:C)-induced inflammation via TLR3 pathway suppression in HDF cells

**Fig. 4:**
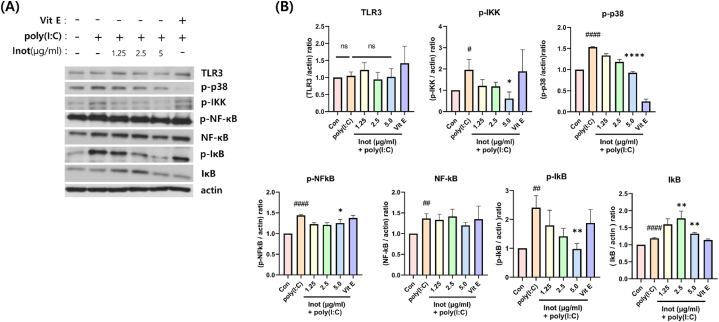
Reduction of TLR3 inflammatory signaling pathway protein by inotodiol in poly(I: C)-induced inflammation model. HDF cell was pre-treated Inotodiol dose-dependently with serum-free media 5 h and incubated poly(I:C) 10 μg/ml for 24 h and protein Lysis for Western blot analysis (A) Represent data with indicated phospho-antibodies, and TLR3, NFκB, IκB and actin antibodies. (B) Relative protein expression was quantified using Image J. data are presented in mean values ± SD. #p < 0.05; ##p < 0.005; ####p < 0.0001 vs. the control group. *p < 0.05; **p < 0.005; ****p < 0.0001 vs. the poly(I:C) group.

To investigate the effect of inotodiol, we evaluated the expression of the NF-κB transcription factor, which induces the expression of pro-inflammatory genes. In HDF cells with poly(I:C)-induced inflammation, after 5 h of pre-treatment with inotodiol, it was observed that *p*-IKK and *p*-IκB levels decreased in an inotodiol dose-dependent manner (Fig. 4A and B). In addition, the expression of TLR3 was slightly increased by poly(I:C) treatment, while it was decreased by pre-treatment with inotodiol. This finding indicates that inotodiol prevents inflammation by inhibiting and regulating the TLR3 signaling pathway induced by poly(I:C) (Fig. 4).

## 4 Discussion

In this study, the anti-inflammatory effect of inotodiol was demonstrated in poly(I:C)-induced inflammation in HDF. An inflammation model using poly(I:C) stimulation in HDF was also established (Fig. 1, D). The TLR3 signaling pathway through poly(I:C) stimulation is well-known and is mediated by NF-kB. Therefore, to evaluate the anti-inflammatory effects of inotodiol, we investigated the gene expression of inflammatory cytokines regulated by the NF-κB pathway (Fig. 2). Furthermore, the secretion of inflammatory cytokines was evaluated (Fig. 3). In addition, to further investigate how inotodiol mediates the anti-inflammatory effect, the expression level and phosphorylation of signaling proteins were investigated. Inotodiol protects against inflammation induced by poly(I:C) stimulation (Fig. 4).

In this study, the inflammatory pathway mediated by the NF-kB and TLR3 pathways was investigated. In addition, a previous study reported that procollagen was decreased by poly(I:C) in human skin fibroblasts, but our results showed an increased pattern in contrast to other reports. It is considered that the primary cell origin probably causes the difference between the foreskin and dermal skin [25]. Furthermore, the amount of procollagen secreted from fibroblasts was increased following inotodiol treatment independent of poly(I:C) stimulation (Fig. S2). This result may be attributable to a regenerative effect owing to the autocrine or paracrine phenomenon caused by the expression of IFNs.

## 5 Conclusions

**Fig. 5:**
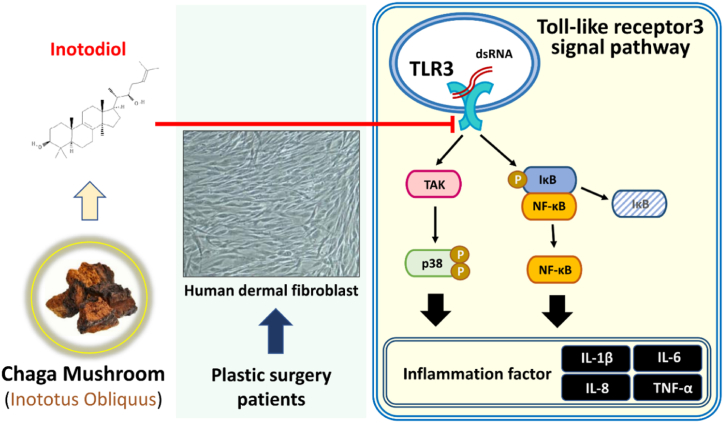
The preventative effect of inotodiol on inflammation from dsRNA exposure in HDF. A schematic representation of inotodiol suppression effect on poly(I:C)-induced inflammation through TLR3 pathway in HDF.

This study suggests that inotodiol may prevent dsRNA-induced inflammation via the TLR3 signal pathway in HDF (Fig. 5). Therefore, we propose the possibility of using inotodiol as a functional cosmetic material with anti-inflammatory and skin elasticity improvement properties.

## Institutional Review Board statement

The Ethics statement was approved by the Institutional Review Board (IRB) of Chungnam National University Hospital (IRB No: 2020-11-001) and the study was performed according to the approved protocol.

## Informed consent statement

Not applicable.

## Data availability statement

The data presented in this study are available in the article and supplementary material.

## Declaration of competing interest

The authors declare that they have no known competing financial interests or personal relationships that could have appeared to influence the work reported in this paper.
