# Supplementary material for: Preventive effects of inotodiol on polyinosinic–polycytidylic acid-induced inflammation in human dermal fibroblasts
Source: Heliyon. 2023 Oct 10;9(10):e20556. doi: 10.1016/j.heliyon.2023.e20556 (PMC10597809; doi:10.1016/j.heliyon.2023.e20556)
Supplement: Multimedia component 1 [file mmc1.docx]

**Supplementary figure legend and figure**

**Figure.S2 Inotodiol pretreatment enhanced procollagen secretion independent in poly(I:C)-induced inflammation in HDF cells.** HDF cell was pre-treated with Inotodiol dose-dependently with serum-free media 5hr and incubated poly(I:C) 10 μg/ml for 24hr and culture media collection and protein lysis for western blot analysis. (A) Shown protein Levels of procollagen-containing media and actin-containing cell lysates

**Fig.S2**

**
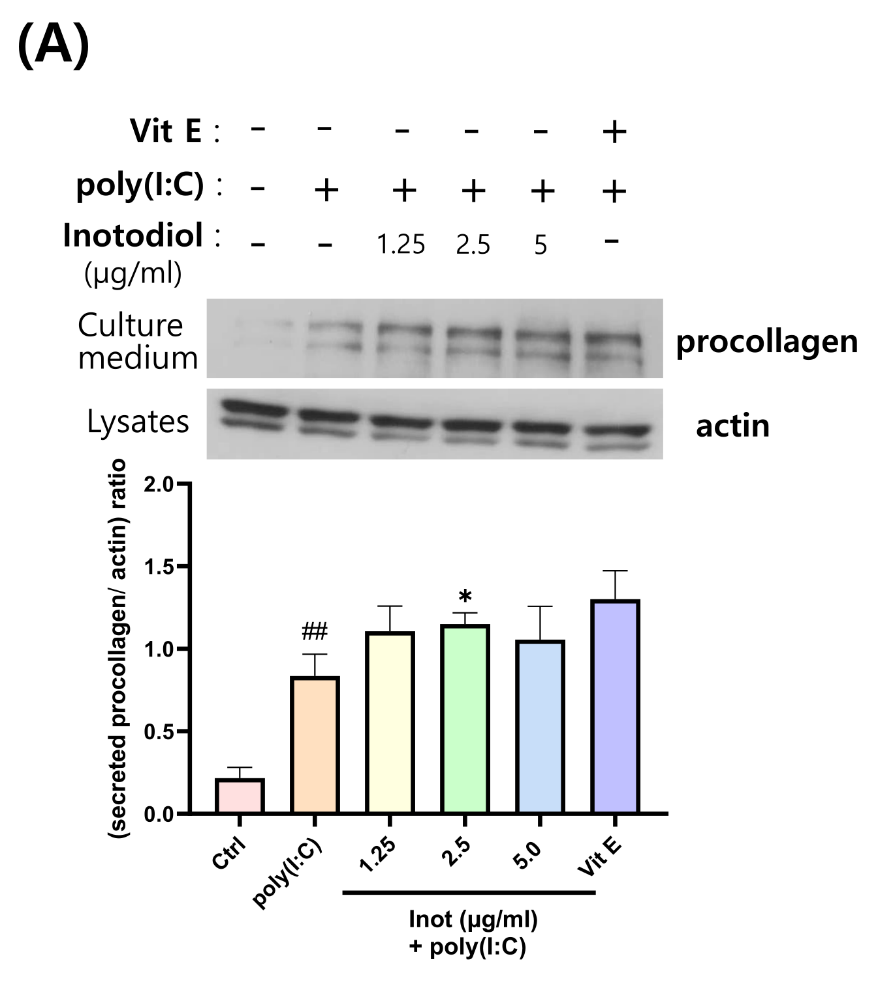
**
